# Analysis on the Temporal and Spatial Features of the Coupling and Coordination of Industrialization and Agricultural Green Development in China during 1990–2019

**DOI:** 10.3390/ijerph18168320

**Published:** 2021-08-06

**Authors:** Hongpeng Guo, Xin Yi, Chulin Pan, Baiming Yang, Yin Li

**Affiliations:** 1 College of Biological and Agricultural Engineering, Jilin University, 5988 Renmin Street, Changchun 130022, China; ghp@jlu.edu.cn (H.G.); yix20@mails.jlu.edu.cn (X.Y.); 2 Changchun Guoxin Modern Agricultural Science and Technology Development Co., Ltd., Shuangyang District, Changchun 130600, China; Ybm0431@163.com (B.Y.); 15243101004@139.com (Y.L.)

**Keywords:** industrialization, agricultural green development, coupling coordination degree, spatial autocorrelation, China

## Abstract

In the past 30 years, China’s industrialization level has developed rapidly, and agricultural green development (AGD) is facing severe challenges. The research on the temporal and spatial features of the coupling and coordination of industrialization and agricultural green development is a key issue to promote the sustainable development of agriculture. This paper takes China’s industrialization and AGD level as the research object, and uses panel data from 31 provinces in China from 1990 to 2019 to construct an evaluation index system for industrialization and AGD. This paper uses the coupling coordination degree model and spatial autocorrelation analysis method to calculate, test and analyze the temporal and spatial features of the coupling coordination level of industrialization and AGD. The results show that: this paper compares the industrialization and AGD levels during the study period and finds that China’s industrialization development level is on the rise as a whole, and the AGD level shows a downward trend first and then rises later. Through the horizontal comparison of different regions, this paper finds that there is a large regional imbalance in the level of industrialization and AGD. The coupling and coordination level of industrialization and AGD has always been primary. From the time point of view, coupling coordination degree shows a trend of first decline and then rise. From a spatial point of view, coupling coordination degree varies greatly among provinces, with the eastern, central and western regions decreasing successively. The level of coupling coordination has obvious positive autocorrelation in spatial distribution, and presents significant spatial agglomeration characteristics in space. The research results can provide a theoretical basis for regionally differentiated governance of the coordinated development of industrialization and AGD, and promote coordinated development.

## 1. Introduction

China’s total GDP ranks second in the world, and it has become the world’s largest newly industrialized country and the second largest economy [1]. “Innovation, coordination, green, openness and sharing” are the main characteristics of China’s current development. However, the contradiction between industry and resource and environmental constraints is still prominent [2]. The cost of environmental resources for agricultural production is too high. At present, crop production is heavily dependent on chemical fertilizers, pesticides, diesel fuel and irrigation. However, excessive use of these inputs will not only lead to increased greenhouse gas emissions [3], water consumption and water pollution [4], it will also cause serious acidification of farmland soil, threaten agricultural production, and seriously hinder AGD.

The transfer of rural surplus labor from the agricultural sector to non-agricultural sectors such as industry is an important way to eliminate the difference between workers and farmers and realize the transformation from traditional agricultural society to modern industrial society or even post-industrial society [5]. The transfer of rural surplus labor from agriculture to industry will increase the welfare of farmers [6]. This is the prerequisite and characterization of industrial development and an important aspect of industrialization. The concept of sustainable development has become a social consensus. The “True green growth” concept aims to reduce the environmental impact of economic growth [7]. The concepts of “Preferring green water and green mountains, not gold mountains and silver mountains” and “Green water and green mountains are golden mountains and silver mountains” are deeply rooted in the hearts of the people. Agriculture is the core of sustainable development. It determines the relationship between the global economy, society and the natural world [8]. AGD is gradually integrated into all aspects of economic and social development. Facts have proved that industrialization and AGD have become the driving force and index of national economic development and social transformation. The systematic analysis of the development level of industrialization and AGD, as well as the coupling and coordination of the two systems, and the temporal and spatial features can provide a scientific reference for national and regional development decisions.

## 2. Literature Review

There are few studies on the coordinated relationship between industrialization and AGD in the existing literature, but scholars have done a lot on industrialization and agricultural development or ecological environment. The concept of “AGD” has been fully proposed in a relatively short time. Scholars’ specific research on the AGD is relatively rare. However, related “agricultural sustainability” research is relatively rich, which can provide a reference for studying the relationship between China’s industrialization and AGD.

### 2.1. Research on the Relationship between Industrialization, Agricultural Development, and Ecological Environment

Industrialized economies tend to grow faster, while non-industrialized economies lag behind [9]. Industrialization is a rapid and long-term process of comprehensive changes in the economic structure, a process of improving the quality and efficiency of economic growth, and a historical process from an agricultural society to an industrial society. With the update and iteration of new technologies, the conditions for industrial development are gradually changing, and the conditions for agricultural development will also change with the advancement of biotechnology. The process of technological change is related to the increase in labor productivity brought about by the use of fertilizers and mechanization, together with the law of centralized agricultural land ownership, releasing cheap labor for industry [10]. In short, the high output of agriculture can accelerate the process of industrialization, while the lag of industrial production will hinder the development of agriculture [11]. At the same time, in the process of industrialization and agricultural development, the role of local governments cannot be ignored. The response of local governments to changes in national development policies will affect the development of local societies and rural microregions [12]. As long as external financial resources have appropriate goals and effective use, conditions can be created for the sustainable development of the economy [13]. Therefore, it is necessary for the government to consider the relationship between agricultural development and industrialization before introducing new agricultural policies [14]. The environmental protection of agricultural ecology is currently a major strategic task for the sustainable development of agriculture and rural areas in China. Human activities have significantly changed the natural ecosystem [15]. This seriously threatens biodiversity [16]. For example, the expansion of industrialization has caused a large amount of agricultural land loss and environmental pollution [17]. The exploitation of natural resources will also have a negative impact on the environment [18], which will also have a greater impact on the improvement of agricultural land and the quality of agricultural products. International policy makers are gradually paying attention to issues of green growth, the promotion of energy efficiency and clean energy technology, and sustainable development [19].

### 2.2. Research on the Sustainable Development of Agriculture

After the Second World War, due to the blind pursuit of the efficiency of agricultural industrialization, agricultural development continued to suffer from problems such as overproduction, biodiversity and loss of soil fertility [20]. Modern agriculture must meet new challenges, such as producing healthy food, adapting to climate change [21], while meeting the needs of a growing population [22] protecting natural resources and protecting landscapes [21]. Scholars began to carry out a series of studies on sustainable agricultural development in response to this issue.

Sustainable development in agriculture refers to agricultural activities that are environmentally sound, technically appropriate, economically feasible and socially acceptable. At the same time, the realization of sustainable agriculture requires the optimization of the agricultural system, including the coordination and trade-offs of a series of factors such as agronomy, environment, social economy, agricultural product diversity, and human nutrition [8]. Sustainable agriculture integrates biology, chemistry, physics, ecology, economics, and social sciences in an integrated manner to develop new agricultural practices that are safe and will not damage the environment [23]. It is a choice to solve the resource and environmental constraints faced by food production through ecological methods [24].

Research on the comprehensive evaluation of sustainability includes social, economic and ecological dimensions, and goes beyond the current linear evaluation methods [25]. Rodrigues et al. (2018) evaluated the impact of ecological intensification measures on coconut production, and the results showed that diversified technologies are positively correlated with higher agricultural sustainability [26]. Buseth (2019) focuses on cross-disciplinary research on green economy, political economy, ideology, etc. [27]. Luis (2021) discusses sustainable development goals and indexes [28]. Nadaraia (2021) identified 47 indexes that can be used to assess the sustainability of plantation agricultural systems [29]. Wang et al. (2021) analyzed the sustainable development of agriculture from the three dimensions of environment, society and economy, identified the main factors affecting the sustainable development of agriculture, and proposed improvement measures and management suggestions to reduce obstacles to sustainable agricultural development and improve Sustainable Agriculture Practice [22].

### 2.3. Research on Agricultural Green Development

Due to the uncertainty brought about by environmental changes, green development is receiving increasing attention from countries all over the world [30]. Green growth is more conducive to the economy, the environment, balance and sustainability. Generally speaking, the concept of green development is considered the second generation of the concept of sustainable development [31]. Wang et al. (2021) used the entropy TOPSIS model and Theil index to reveal the differences in the green development of industries in China’s three major urban agglomerations [32]. The overall goal of AGD is to coordinate “green” and “development” to realize the transformation of current agriculture with high resource consumption and high environmental cost to a green agriculture with high productivity, high resource utilization efficiency and high efficiency [33]. Among them, the research on AGD mainly focuses on evaluating the process of AGD by constructing an AGD index [34,35]. Wang et al. (2021) used the Slacks-based model to measure the efficiency of agricultural green production in the middle reaches of the Yangtze River based on the input-output index system of agricultural green production [36].

### 2.4. Research Review

In summary, with the increasing number of research dimensions, the research content has evolved from a single study of the relationship between industrialization and AGD to the relationship between different research objects, and the research methods have evolved from methods such as the analytic hierarchy process and entropy method to the Tobit model and the degree of coupling and coordination. Complicated evaluation methods such as models. The depth of theoretical analysis continues to increase, and research is closely related to national and regional development strategies. It has become an important reference for national and local governments’ scientific decision-making. However, China has a vast territory, and there are obvious regional differences in natural geographic background and economic development level. Scholars’ research on the level of national industrialization, agricultural green development and the degree of coupling and coordination between the two systems is still blank. This paper draws on the methods of the above research. This paper intends to construct a set of index systems that can comprehensively and scientifically reflect the level of “industrialization and green agricultural development”. The research object of this paper is the level of industrialization and AGD in 31 provinces (autonomous regions and municipalities) in China. Based on the panel data from 1990 to 2019, this paper uses the improved entropy method to determine the index weight, and uses the coupling coordination degree model to calculate the coupling coordination degree of industrialization and AGD. This paper uses ArcGIS spatial analysis to reveal its spatial differences, and combines spatial autocorrelation analysis methods to test and analyze the temporal and spatial features of the coupling and coordination level of industrialization and AGD. This paper hopes to provide theoretical reference and policy guidance research content for the subsequent coordinated development of China’s industrialization and AGD.

## 3. Materials and Methods

### 3.1. Data Sources

**Figure 1:**
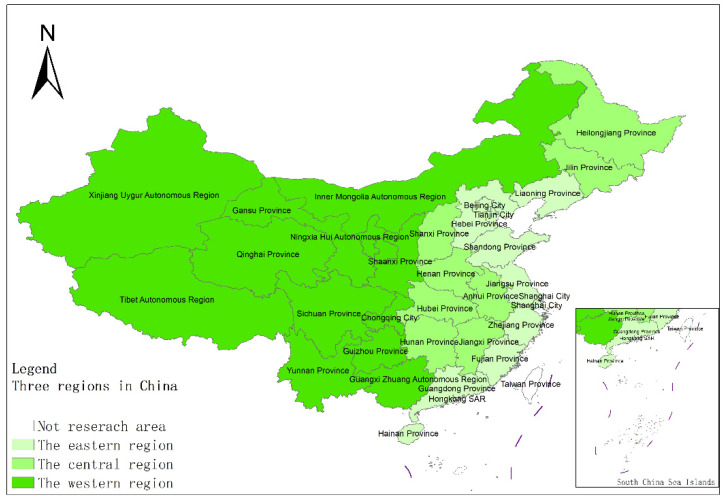
The spatial distribution features of China’s three regions. Source: [34].

The data required for this paper are from the 1991–2020 China Statistical Yearbook, China Industrial Statistical Yearbook, China Rural Statistical Yearbook, China Regional Economic Statistical Yearbook, as well as the statistical yearbooks of various provinces, autonomous regions, and municipalities, economic and social development statistical bulletin. Due to the availability of data, Hong Kong, Macau and Taiwan have not been included in the study. China has a vast territory, and there are obvious differences in agricultural resource endowments and support policies. This paper analyzes the spatial differences of industrialization, AGD levels and coupling and coordination features in different regions, and divides China’s 31 provinces (autonomous regions and municipalities) except Hong Kong, Macau, and Taiwan into three major regions: the east, the middle, and the west. The specific division is shown in Figure 1.

### 3.2. Index Construction

#### 3.2.1. Industrialization Development Level Evaluation Index

China’s industrialization is one of the characteristics of economic growth [37]. The industrialization development level evaluation indexes select four indexes: per capita GDP [1], the proportion of secondary industry output [38], the proportion of secondary industry employment [39], and secondary industry’s labor productivity [32,39]. This paper conducts a comprehensive evaluation of the level of industrialization development from the perspective of economic strength, output, employment, and labor productivity.

#### 3.2.2. Evaluation Index of Agricultural Green Development Level

Although domestic research on the level of AGD are abundant, existing studies are not uniform in the index system for evaluating the level of AGD. China’s agricultural sector formally proposed the goal of “one control, two reductions and three basics” in 2015. “One control” refers to controlling the total amount of agricultural water use and agricultural water environmental pollution, ensuring that the total amount of agricultural irrigation water is maintained at 372 billion cubic meters. The water quality of farmland irrigation meets the standard. “Two reductions” refers to the reduced use of chemical fertilizers and pesticides. The “three basics” means that livestock and poultry manure, agricultural film, and crop straw are basically recycled, comprehensively recycled, and treated in a harmless manner [40]. On the whole, the amount of manure produced in China’s livestock and poultry farming has little impact on the environment [41]. Farms above the designated size break up the straw and return it to the field [42] or carry out conservation tillage. Moreover, this paper is affected by the availability of data, and the harmless treatment rate of livestock and poultry manure and straw has not been included in the evaluation index of the level of AGD. The recycling rate of mulch film is low [43], so the intensity of use is used. This paper selects eight indexes to evaluate the level of AGD: the per capita disposable income of rural residents [34,40], the level of agricultural mechanization [39,40,44] and the rate of land output [29,45], the level of farmland being irrigated [39,44], the intensity of fertilizer used [29,44], the intensity of pesticides used [29], the intensity of the used agricultural film [46], the proportion of disaster area [34]. The evaluation index system is shown in Table 1.

### 3.3. Analytical Method

#### 3.3.1. Entropy Weight Method

In order to effectively avoid subjectivity [47], this paper uses the entropy method to determine the index weight. The smaller the information entropy, the greater the index weight [48]. This paper selects the data as panel data. In order to make the years comparable, time variables are introduced to improve the entropy method [34]. The evaluation model is as follows:

1. Construct the original index matrix data: given years, provinces, indexes, the original index matrix is $X={\left\{x_{\lambda ij}\right\}}_{h*n*m}\left(1\leq \lambda \leq h,1\leq i\leq n,1\leq j\leq m\right)$, and $x_{\lambda ij}$ is the index value of the λ year, the i province, and the j index. In this paper h, n and m are 30, 31 and 8.
2. Dimensionless processing of the range standard method for each index in the index system:
  (1) $$\mathrm{Standardization}\;\mathrm{of}\;\mathrm{positive}\;\mathrm{indexes}:\;Z_{\lambda ij}=\frac{x_{\lambda ij}-x_{min}}{x_{max}-x_{min}}$$
  (2) $$\mathrm{Standardization}\;\mathrm{of}\;\mathrm{negative}\;\mathrm{indexes}:\;Z_{\lambda ij}=\frac{x_{max}-x_{\lambda ij}}{x_{max}-x_{min}}$$
3. Determine the index weight:
  (3) $$P_{\lambda ij}=\frac{Z_{\lambda ij}}{\overset{h}{\underset{\lambda =1}{\sum }}\overset{n}{\underset{i=1}{\sum }}Z_{\lambda ij}}$$
4. Calculate the entropy value of various indexes:
  (4) $$E_{j}=-k\overset{h}{\underset{\lambda =1}{\sum }}\;\overset{n}{\underset{i=1}{\sum }}P_{\lambda ij}\ln P_{\lambda ij}$$
  (5) $$k=\frac{1}{\ln\left(hn\right)}$$
5. Calculate the redundancy of the entropy values of various indexes:
  (6) $$D_{j}=1-E_{j}$$
6. Calculate the weight of each index:
  (7) $$W_{j}=\frac{D_{j}}{\sum_{j=1}^{n}D_{j}}$$
7. Construct a multi-index weighted comprehensive evaluation model:
  (8) $$U=\overset{n}{\underset{j=1}{\sum }}W_{j}Z_{\lambda ij}$$

#### 3.3.2. Coupling Coordination Degree Model

Coupling refers to the process in which the systems under study influence each other through different links [49], Coupling coordination degree model is an evaluation of the degree of mutual influence between systems. This model has been widely used in the fields of social economics, biology, agriculture, ecology, etc. [31]. The two systems of China’s industrialization and AGD do not exist independently, and there are mutual promotion and mutual constraints between them. This paper uses the coupling coordination degree model to express the industrialization index $\left(U_{1}\right)$ and the AGD index $\left(U_{2}\right)$. This paper first calculates the coupling degree (C) of each province (autonomous regions, municipalities) from 1990 to 2019. On this basis, this paper calculates the coupling coordination degree of the two systems [50] (D). The value range of D is (0,1). Specifically, the closer to 1, the more coordinated and balanced the industrialization and AGD; the closer to 0, the worse the coordination and imbalance of development. The calculation formula is:

(9) $$C=2{\left[\frac{\left(U_{1}\cdot U_{2}\right)}{\left(U_{1}+U_{2}\right)}\right]}^{\frac{1}{2}}$$

(10) $$T=\alpha U_{1}+\beta U_{2}$$

(11) $$D=\sqrt{C\cdot T}$$

In the formula, T is the comprehensive coordination index of industrialization and AGD; α and β are undetermined coefficients α+β=1. In this paper, an improved entropy method is used to determine the value of the undetermined coefficient, α=0.3332, β=0.6668. Referring to related literature [51], the coupling and coordination level of industrialization and AGD in various provinces (autonomous regions and municipalities) is divided into 10 levels: extreme disorder (0, 0.1), severe disorder (0.1, 0.2), moderate disorder (0.2, 0.3), mild disorder (0.3, 0.4), on the verge of disorder (0.4, 0.5), grudging coordination (0.5, 0.6), primary coordination (0.6, 0.7) intermediate coordination (0.7, 0.8), good coordination (0.8, 0.9), quality coordination [0.9,1].

#### 3.3.3. Spatial Autocorrelation Analysis

As China’s industrialization and AGD have different degrees of impact on the neighborhood in terms of spatial distribution features, this paper uses the establishment of a spatial autocorrelation analysis model to study the changes in the spatial layout of the coupling and coordination of industrialization and AGD. At present, global spatial autocorrelation and local spatial autocorrelation are the two systems most commonly used in exploratory spatial data analysis [52]. Among them, the global spatial autocorrelation reflects the spatial correlation mode of a certain attribute value in the entire research area. The local spatial autocorrelation further reveals the correlation and distribution law of certain attribute values in the local space. The global spatial autocorrelation is usually represented by the global Moran’s I measurement, which ranges from [−1−1]. When Moran’s I<0, it means that there is a negative spatial correlation between industrialization and AGD; when Moran’s I=0 it means that there is no spatial correlation; when Moran’s I>0, there is a positive correlation. The calculation formula is [53]:

(12) $$\mathrm{Moran}’s\;I=\frac{n\sum_{i=1}^{n}\;\sum_{j=1}^{n}\;W_{ij}\left(x_{i}-\bar{x}\right)\left(x_{j}-\bar{x}\right)}{\sum_{i=1}^{n}\;{\left(x_{i}-\bar{x}\right)}^{2}\sum_{i=1}^{n}\;\sum_{j=1}^{n}\;W_{ij}}$$

In the formula, n is the number of research areas, $X_{i}$ and $X_{j}$ represent the index values of areas i and j; $W_{ij}$ represents the proximity relationship between areas i and j, when $W_{ij}=1$, the area i and j are adjacent, when $W_{ij}=0$, the area i and j are not adjacent.

Local autocorrelation index $G^{*}$ can detect whether there are high address clusters in a local area. The calculation formula is:

(13) $$G^{*}=\frac{\sum_{j=1}^{n}\;W_{ij}X_{j}}{\sum_{i=1}^{n}\;X_{i}}$$

In the formula, $G^{*}$ is the local autocorrelation index.

## 4. Results

### 4.1. Index Weight

In this paper, Equations (1)–(7) are used to determine the index weights, and the weight results are shown in Table 2.

### 4.2. China’s Industrialization and Agricultural Green Development

#### 4.2.1. Industrialization Development Index

After determining the weights, Formula (8) is used to obtain the industrialization development index of China’s 31 provinces (autonomous regions and municipalities). The results are shown in Table 3. The level of industrialization development of China’s provinces (autonomous regions and municipalities) is on the rise as a whole, showing a spatial trend of the east > the middle > the west. Areas with higher development indexes than the average are mainly located in the eastern coastal areas of Bohai Economic Rim, Yangtze River Delta Economic Zone, Pearl River Delta and Beijing-Tianjin-Hebei region have relatively developed economies. Regions with a development index below the average are mainly distributed in Southwest and Northwest Territories, which is basically the same as the regional economic development pattern.

#### 4.2.2. Agricultural Green Development Index

Using the same method to obtain the AGD index of China’s 31 provinces (autonomous regions and municipalities), the results are shown in Table 4, the overall AGD level of China’s provinces (autonomous regions and municipalities) has declined first and then increased. From a spatial perspective, there is a trend of east > middle > west. Areas with an AGD index higher than the average are mainly distributed in the North China Plain, southeastern coastal areas and Qinghai-Tibet plateau. Areas with an index lower than the average are mainly distributed in the Yunnan-Guizhou-Sichuan, Shaanxi-Gansu-Ningxia regions. The regional pattern may be related to the regional social economy The level of development is related to the background of natural geography, etc.

#### 4.2.3. Overall Development Level

**Figure 2:**
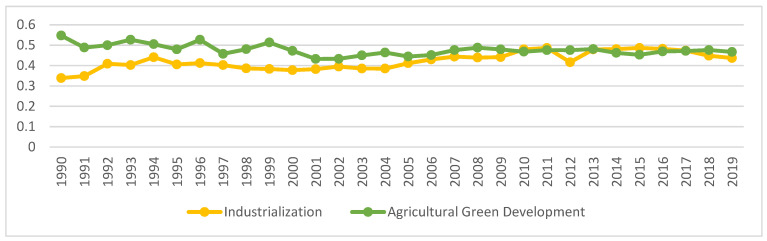
China’s industrialization and agricultural green development level 1990–2019. Data Source: The author calculated based on China Statistical Yearbook, China Industrial Statistical Yearbook, China Rural Statistical Yearbook, China Regional Economic Statistical Yearbook.

Using the provincial average of each index, explore the difference between China’s industrialization and AGD index from 1990 to 2019, as shown in Figure 2.

From 1990 to 2019, China’s industrialization development has generally gone through four development stages. Industrialization was in a rapid development stage from 1990 to 1994; the industrialization index was stagnant from 1994 to 2004; the industrialization index was fluctuating and rising after 2004–2015; the industrialization index was declining during 2015–2019. The industrialization development index remained at about 0.42 during the study period. The level of AGD is more complicated. From 1990 to 2001, the AGD index was in a state of volatility and decline; from 2001 to 2015, the AGD index was in a trend of first increasing and then decreasing; 2015–2019 AGD index was in a state of volatility and rising During the research period, the AGD index remained at around 0.47.

### 4.3. Coupling and Coordination Degree of Industrialization and Agricultural Green Development

**Figure 3:**
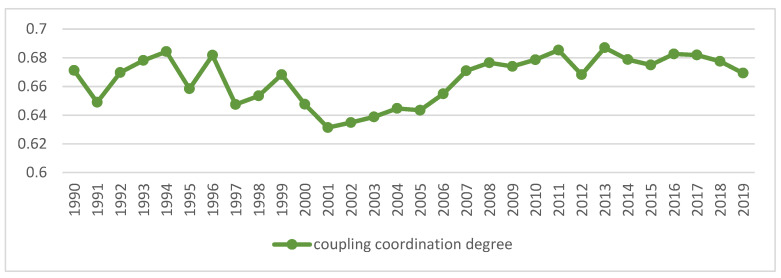
Trends of coupling coordination of China’s industrialization and agricultural green development. Data Source: the authors calculated based on China Statistical Yearbook, China Industrial Statistical Yearbook, China Rural Statistical Yearbook, China Regional Economic Statistical Yearbook.

According to Equation (9)–(11), the degree of coupling and coordination between China’s industrialization and AGD from 1990 to 2019 is calculated, and the calculation results are shown in Figure 3.

On the whole, from 1990 to 2019, the average value of the coupling coordination degree of China’s industrialization and AGD was 0.67, which has been in the primary coordination stage and still has huge development potential. The two systems present a relatively obvious interconnection and mutual influence relationship, and form an open and complex system. In the process of economic development, industrialization and AGD work together, and the disorderly development of any index will lead to a decline in the degree of system coupling and coordination. The coupling and coordination level of the two systems is improving, but the speed of improvement is gradually slowing down, and no further breakthroughs have been made in recent years, and it has entered a bottleneck period of transition to a higher level.

**Figure 4:**
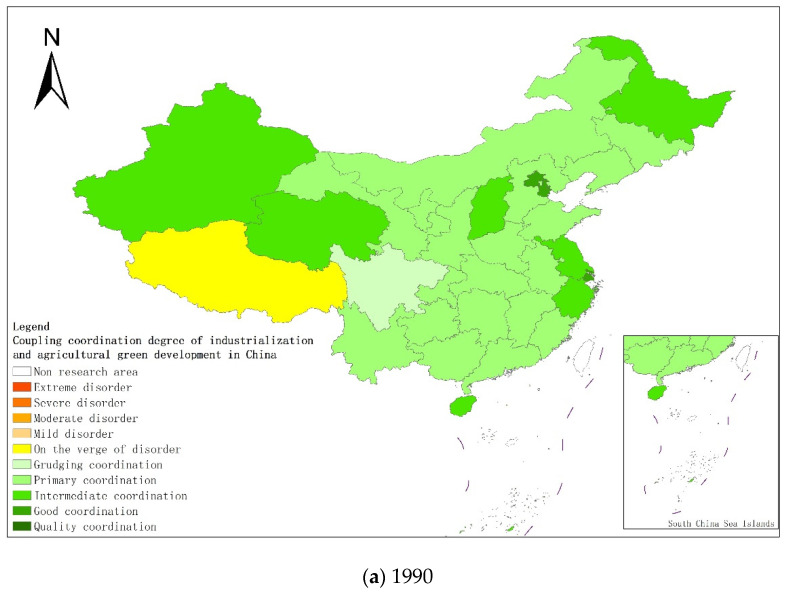

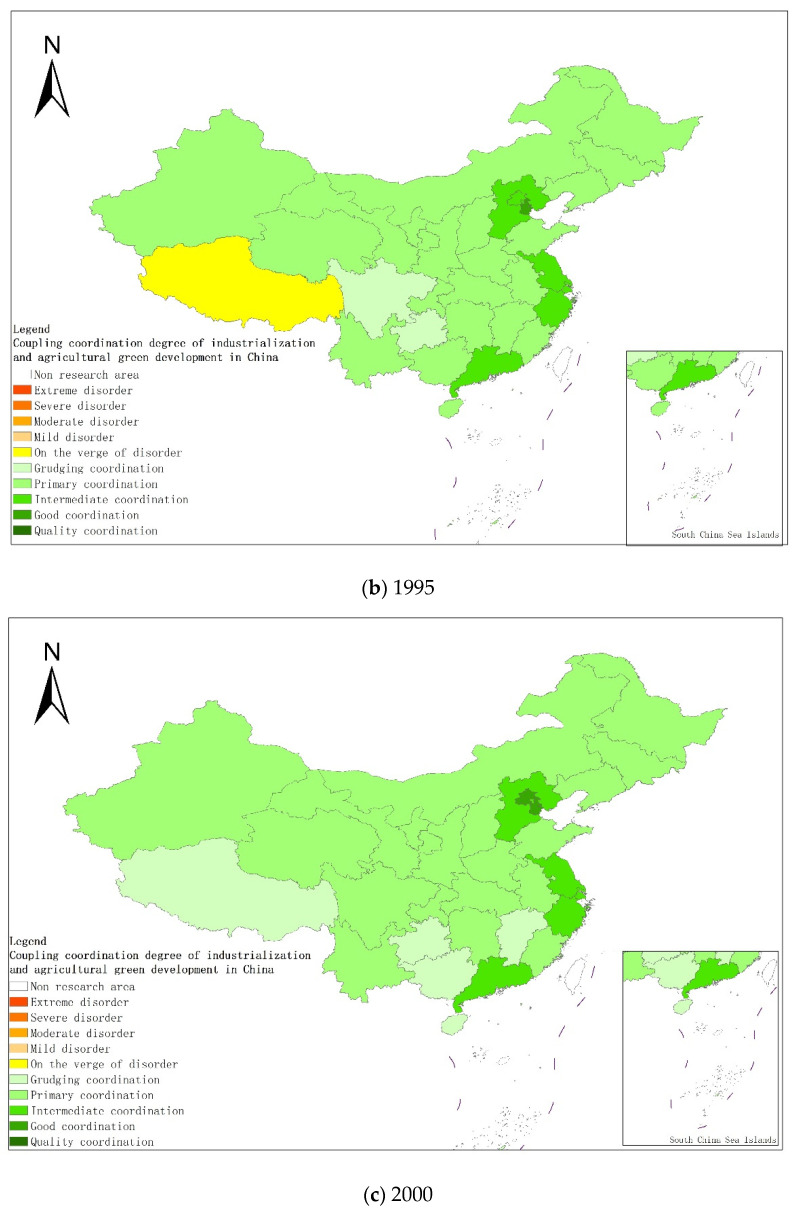

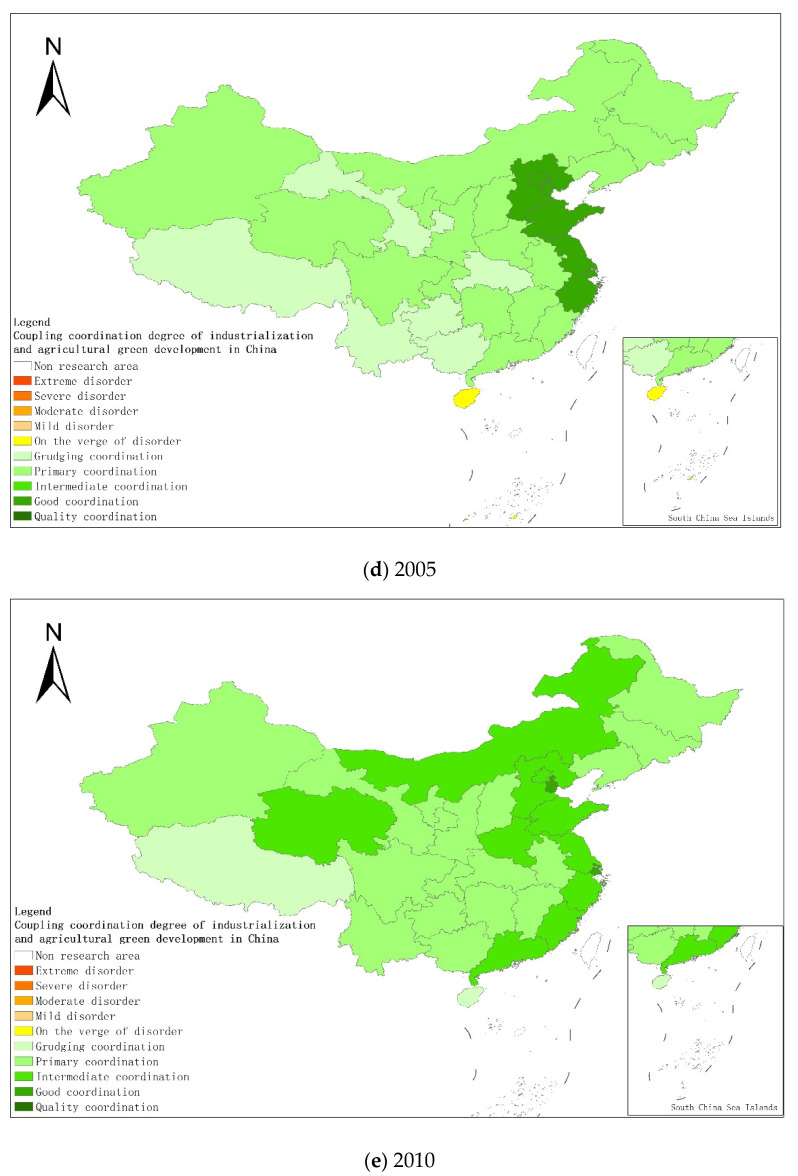

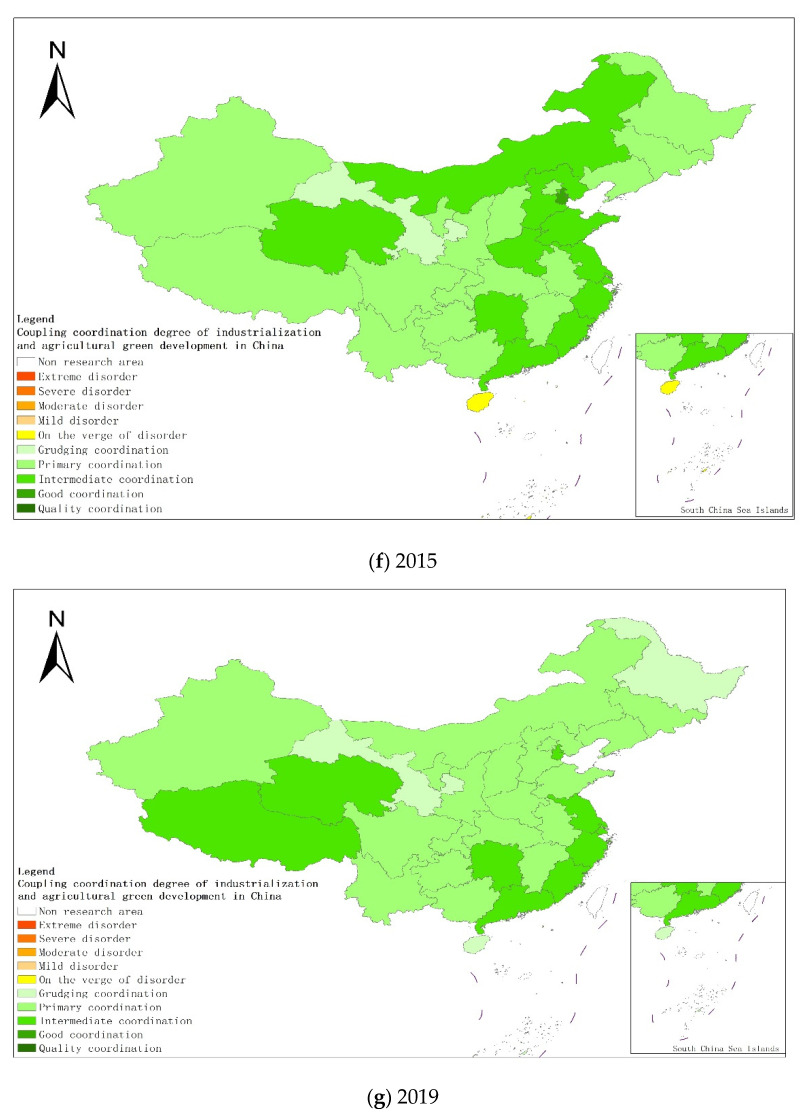
Coupling and coordination types of China’s industrialization and agricultural green development in some years (**a**–**g**). (**a**) 1990; (**b**) 1995; (**c**) 2000; (**d**) 2005; (**e**) 2010; (**f**) 2015; (**g**) 2019. Data Source: the authors calculated based on China Statistical Yearbook, China Industrial Statistical Yearbook, China Rural Statistical Yearbook, China Regional Economic Statistical Yearbook.

From a regional perspective, Figure 4a–g shows the types of coupling coordination in various provinces (autonomous regions and municipalities) in some years. The number of regions on the verge of dysfunction has been reduced from 1 to 0, and no regions are in lightly coordinated and high-quality coordinated provinces. It can be seen from the figure that during the study period, the three major regions showed a trend of first decreasing and then increasing, with the eastern, central and western regions successively decreasing. The eastern region has a relatively high level of industrialization development, but the level of AGD is relatively low. For example, in Fujian, the road of industrialization at the expense of AGD has restricted the further improvement of the level of coupling and coordination. Although the level of industrialization in the central and western regions is not as high as that in the eastern regions, the industrial structure in some regions is reasonable, and the reduction of chemical fertilizers and pesticides has been significant, making the gap in the level of coupling and coordination of industrialization and AGD not obvious.

### 4.4. Spatial Autocorrelation Analysis of Coupling Degree of Industrialization and Agricultural Green Development in China

#### 4.4.1. Global Autocorrelation Test

This paper uses the global spatial autocorrelation analysis method to analyze the degree of coupling and coordination of industrialization and AGD in the study area and make a Moran scatter diagram. Table 5 shows the calculation results of the global Moran index of the coupling coordination degree of China’s industrialization and AGD. Moran indexes are all greater than 0, and the adjoint probability *p* values are all less than 0.1. It shows that the degree of coupling and coordination of China’s provincial industrialization and AGD has an obvious positive autocorrelation in the spatial distribution. That is, the degree of coupling and coordination of China’s provincial industrialization and AGD shows significant spatial agglomeration characteristics in space.

#### 4.4.2. Local Correlation Test

**Figure 5:**
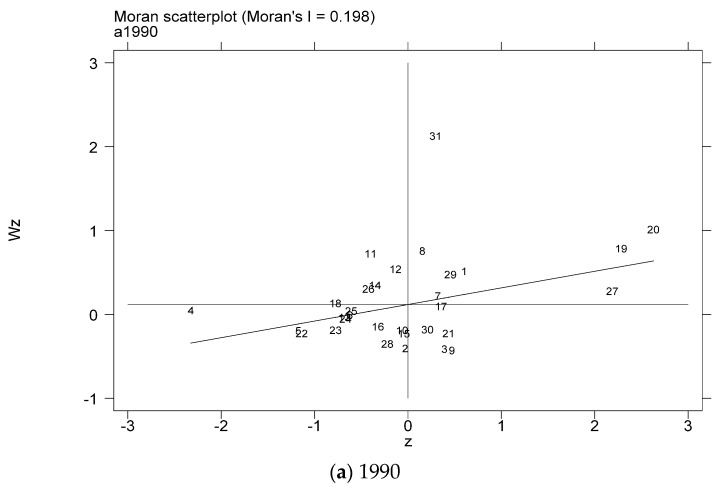

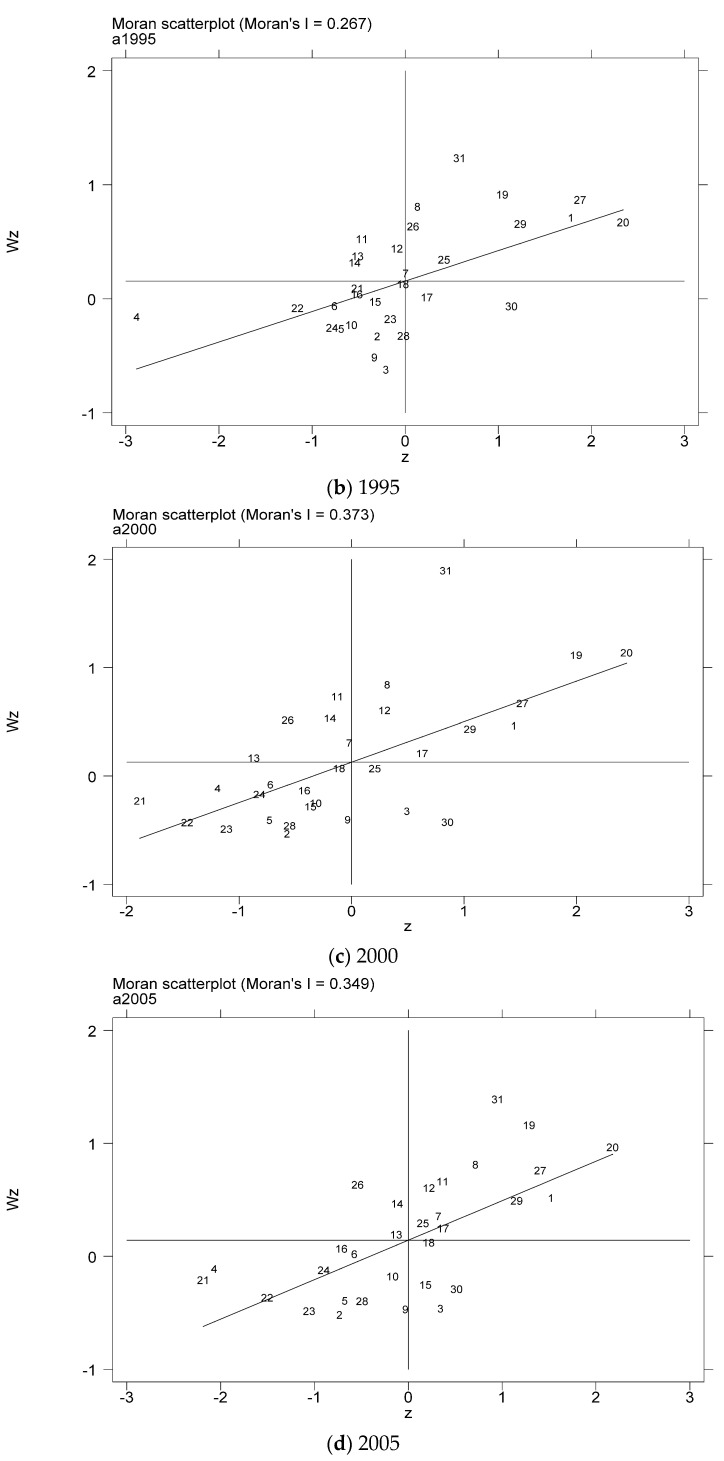

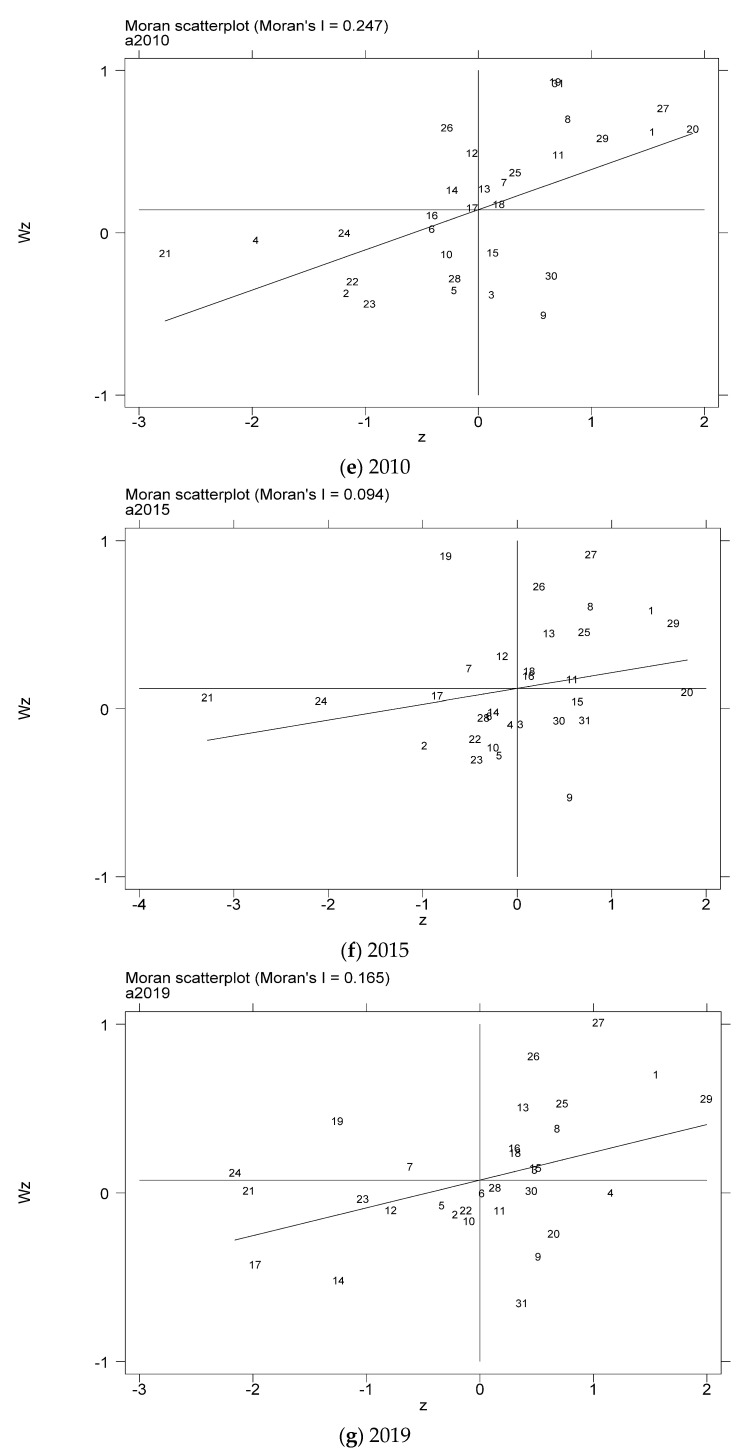
Scatter plot of local Moran index of the coupling coordination degree of China’s industrialization and agricultural green development in some years (**a**–**g**). (a) 1990; (**b**) 1995; (**c**) 2000; (**d**) 2005; (**e**) 2010; (**f**) 2015; (**g**) 2019. Data Source: the authors calculated based on China Statistical Yearbook, China Industrial Statistical Yearbook, China Rural Statistical Yearbook, China Regional Economic Statistical Yearbook.

In order to further test the spatial relationship of the coupling coordination degree of China’s provincial industrialization and AGD, considering the distribution characteristics of the coupling coordination degree of the local area, this paper draws the local Moran index scatter plots for some years based on the inverse distance spatial weight matrix, as shown in Figure 5a–g. The Moran scatter diagram is presented by dividing the space into four quadrant units. The first quadrant is the high-high (H-H) agglomeration area, which means that provinces with a high level of coupling and coordination of industrialization and AGD are surrounded by high-value areas. The second quadrant is the low-high (L-H) agglomeration area, which means that provinces with low levels of industrialization and AGD coupling and coordination are surrounded by high-value areas. The third quadrant is the low-low (L-L) agglomeration area, which means that provinces with a low level of coupling and coordination of industrialization and AGD are surrounded by low-value areas. The fourth quadrant is the high-low (H-L) agglomeration area, which means that provinces with a high level of coupling and coordination of industrialization and AGD are surrounded by low-value areas.

**Figure 6:**
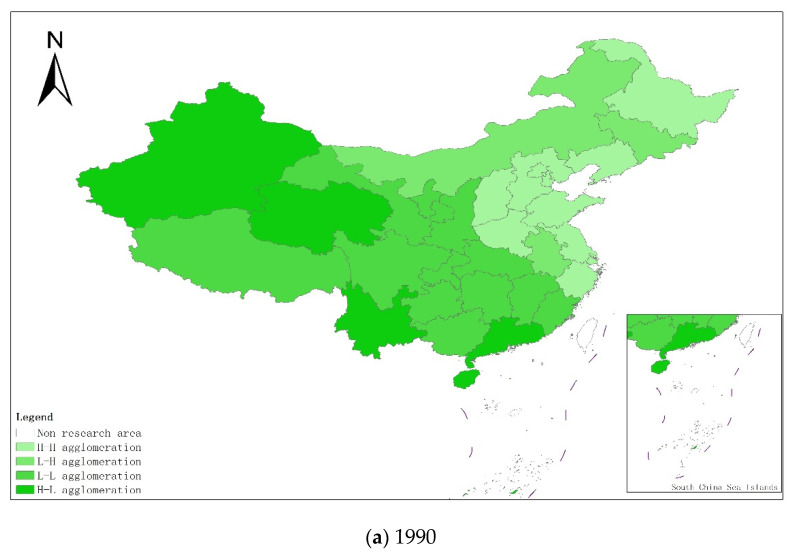

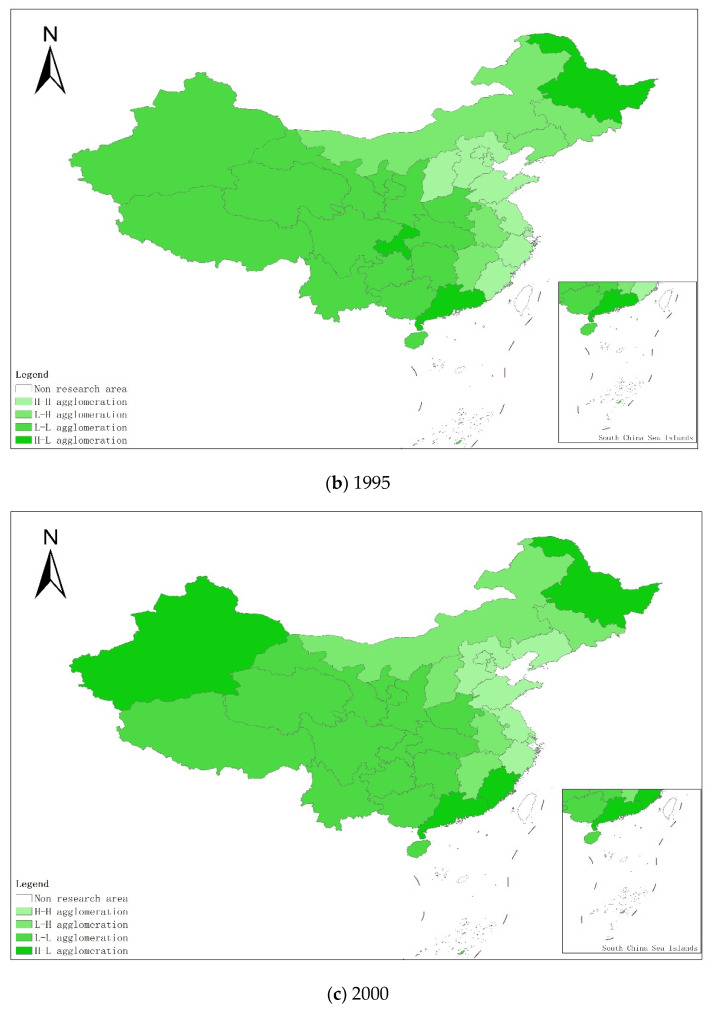

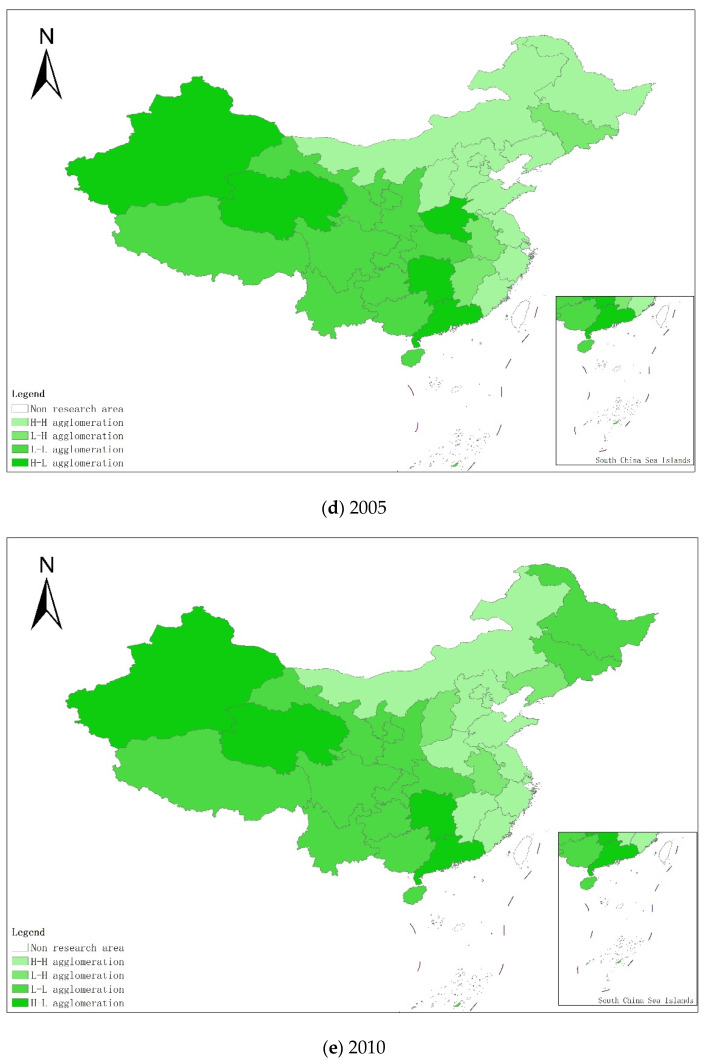

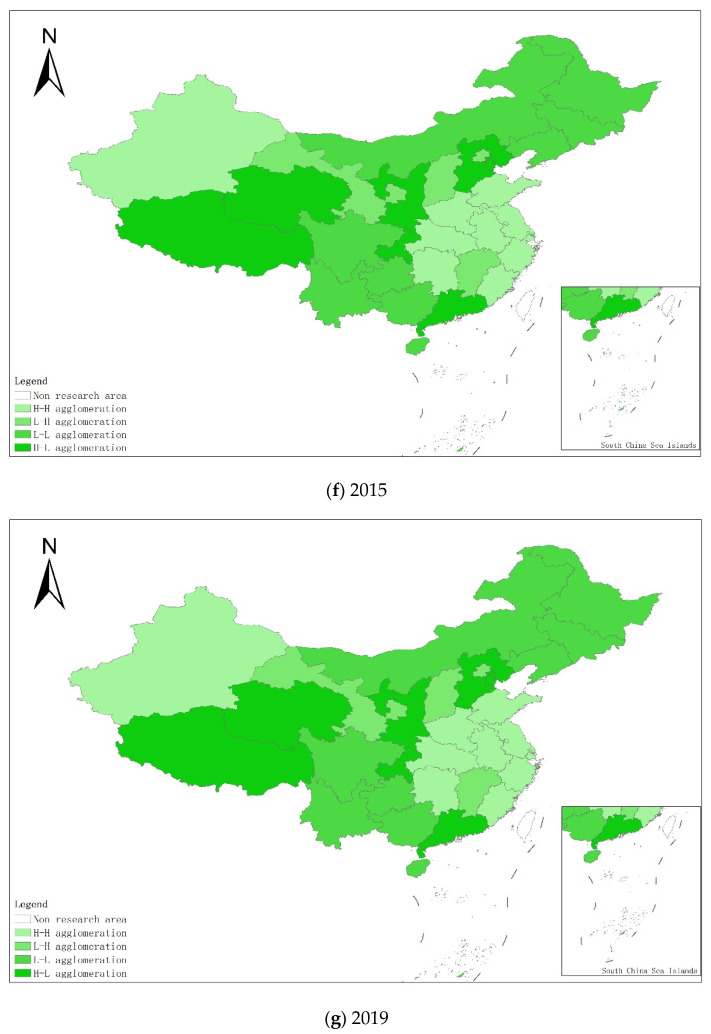
Province distribution of the Moran index of the coupling coordination degree of China’s industrialization and agricultural green development in some years (**a**–**g**). (**a**) 1990; (**b**) 1995; (**c**) 2000; (**d**) 2005; (**e**) 2010; (**f**) 2015; (**g**) 2019. Data Source: the authors calculated based on China Statistical Yearbook, China Industrial Statistical Yearbook, China Rural Statistical Yearbook, China Regional Economic Statistical Yearbook.

In order to more intuitively understand the agglomeration or dispersion of various provinces, the data of each quadrant is summarized and presented in the form shown in Figure 6. From the perspective of time dynamics, the level of AGD in various regions has changed, but in the selected seven years, most of the provinces are located in the H-H agglomeration area and the L-L agglomeration area. In the long run, the provinces located in the quadrants have relatively little change. The provinces located in the H-H agglomeration area for a long time are mostly provinces in the eastern coastal region, while the provinces in the L-L agglomeration area are mostly provinces in the western region. It shows that China’s industrialization and AGD coupling and coordination degree are spatially positively correlated and have high spatial agglomeration.

## 5. Discussion

### 5.1. Characteristics of China’s Industrialization and Agricultural Green Development

From 1990 to 2019, China’s industrialization has generally gone through four stages of development. From 1990 to 1994, industrialization was in a stage of rapid development, and the scale of industrial and economic development was steadily increasing, and the structure was continuously optimized. During 1994–2004, the development of industrialization was at a standstill. During this period, China experienced the huge impact of the Asian financial turmoil and the reform of state-owned enterprises. The major adjustments in the industrial structure led to an increase in the number of laid-off employees in industrial enterprises, which resulted in a low proportion of the employment in the secondary industry. From 2004 to 2015, the industrialization index was in a state of volatility and rising. The level of industrialization maintained a high growth rate, with a growth rate of 26.36%. After economic reform and the process of opening up, the level of industrialization and economic development have been rapidly improved, but this is mostly due to China’s huge “demographic dividend” and resources. The environment has been sacrificed in exchange. After entering the 21st century, facing the problem of increasingly depleted domestic resources and a rapidly deteriorating environment, the previous extensive development model is no longer sustainable. The Chinese government proposes to strengthen the adjustment of industrial structure and increase support for technology-intensive industries, so that a number of high-tech industries that are “high-tech, high-efficiency, and high-efficiency” will develop rapidly. The level of industrialization in 2015–2019 is in a state of decline, and the one-sided pursuit of speed and scale of development has been unable to meet the people’s growing needs for a better life. At the same time, the contradictions between the industrial and agricultural structures accumulated over the past 40 years of economic reform and opening up, the contradictions of uncoordinated regional development, and the contradictions between economic growth and resource and environmental constraints have become increasingly prominent. At the same time, the tertiary industry dominated the economic structure during this period, and the characteristics of post-industrialization were obvious. The proportion of manufacturing output value fell sharply, and the employment of employees continued to transfer to tertiary industry. The Chinese government recognized that economic and social development needs to actively reduce the development speed and improve the internal development quality.

AGD has generally gone through three stages. From 1990 to 2001, the AGD index was in a state of volatility and decline. Since the 1990s, the large-scale use of chemical fertilizers, pesticides, and agricultural films in China’s agriculture has caused serious non-point source pollution. At the same time, the transfer of agricultural surplus labor and the development of agricultural industrialization have been affected by the decline in the level of industrialization during the same period. The above problems have led to a decline in the level of AGD. From 2001 to 2015, the AGD index was in a trend of increasing first and then decreasing. This is because the government attaches great importance to the “three rural” issues, and promulgated the central “No. 1 Document” related to the “three rural” issues in successive years, and issued a series of policies that benefit farmers and strengthen farmers. The large-scale promotion of mechanization in agriculture and the development of industrialization have promoted the enhancement of agricultural disaster resistance. However, the level of irrigated farmland, the low utilization rate of chemical fertilizers and pesticides, and the low recovery rate of agriculture have led to another decline in the level of AGD. From 2015 to 2019, the AGD index was in a state of volatility and rising. As of the end of 2017, the use of pesticides had been reduced for three consecutive years, the use of chemical fertilizers has been reduced for two consecutive years, and the utilization rate of chemical fertilizers and pesticides has increased significantly.

### 5.2. Coordination Level and Spatial Characteristics of China’s Industrialization and Agricultural Green Development

From an overall point of view, the main reason that the coupling coordination level of China’s industrialization and AGD has been in primary coordination is that after more than 40 years of rapid development of economic reform and opening up, China’s economy has gradually entered a stage of new normal development. At this stage, new features different from those of the past appeared, namely, a major change in the economic growth rate, economic structure and the development of economic driving forces. In the new stage of economic development, the level of China’s industrialization and AGD has been undergoing a process from “quantitative change” to “qualitative change”. However, the development from “quantitative change” to “qualitative change” is a gradual and insignificant process. This makes it difficult to achieve a breakthrough in the level of coupling and coordination in a short period of time.

From a regional perspective, from the early stage of economic reform and opening up to around 2000, the Chinese government established the eastern region as a priority growth pole for China’s economy, thereby driving a “goose model” in other regions; from 2000 to 2012, the Chinese government adopted the “Western Development”, regional development strategies such as the “Rise of Central China” and “ Revitalization of the Northeast “ which have gradually promoted balanced regional development; since 2012, all regions have entered a stage of comprehensive and coordinated development. Affected by historical factors, Beijing and Tianjin in the Bohai Rim Economic Circle in the eastern region, and Shanghai, Jiangsu, and Zhejiang in the Yangtze River Delta Economic Circle, have been at a relatively high level of coupling and coordination of industrialization and AGD. These regions have a superior technical level, high market openness and good national support policies, attracting a large number of domestic and foreign enterprises and a large number of rural surplus labor, and the effect of industrial agglomeration is obvious. The improvement of the level of industrialization can understand the improvement of the level of AGD. Therefore, the coordinated development of these regions is at the leading level in the country. Although the central region is not as mature as the eastern coastal regions in terms of technical conditions and market environment, and industrialization has not formed a comparative advantage, these regions are the main agricultural production areas with relatively mature agricultural technology. Moreover, because a large number of rural surplus labor flows to cities in the province and the eastern coastal areas, the level of agricultural industrialization in these areas is relatively high, and the scale effect is obvious. However, the technical level and economic foundation of Guizhou, Tibet, Gansu and other places in the western region are not as superior as those in the eastern region, and the natural conditions are not as good as those in the central region. The AGD model has not yet been formed. As a result, the level of coupling and coordination between industrialization and AGD is low. From this, it is concluded that the level of coordinated development of China’s industrialization and AGD during the study period was an example of primary coordination as a whole, with significant regional differences.

## 6. Conclusions, Limitations and Further Research

Based on a comprehensive understanding of China’s industrialization and AGD level from 1990 to 2019, this paper constructs two systems with 12 indexes, evaluates the industrialization and AGD level, and analyzes the temporal and spatial of the coupling and coordination level of the two systems. feature. The results compare the industrialization and AGD levels during the study period and find that China’s industrialization development level is on the rise as a whole, and the AGD level shows a downward trend first and then rises later. Through the horizontal comparison of different regions, this paper finds that there is a large regional imbalance in the level of industrialization and AGD. The coupling and coordination level of industrialization and AGD has always been in primary coordination. From the time point of view, coupling coordination degree shows a trend of first decline and then rise. From a spatial point of view, coupling coordination degree varies greatly among provinces, with the eastern, central and western regions decreasing successively. The level of coupling coordination has obvious positive autocorrelation in spatial distribution, and presents significant spatial agglomeration characteristics in space.

This paper preliminarily discusses the regional pattern of industrialization and AGD, the characteristics of the coupling coordination level, and the spatial autocorrelation analysis of the coupling coordination level. This paper has some limitations. The connotation of industrialization and AGD is far more abundant than the current index system reflects. Limited by data, it is difficult for this paper to undertake a comprehensive description of industrialization and AGD. In future studies, typical case studies can be conducted on the level of coupling and coordination of industrialization and AGD in different types of regions, in order to find out the factors that affect the level of coupling and coordination of industrialization and AGD, which will help guide the sustainable development of different regions.

## Figures and Tables

**Table 1:** Evaluation index system for the degree of coupling and coordination of industrialization and agricultural green development.

Primary Indexes	Secondary Indexes	Index Description	Units	Direction	Reference
Industrialization	The level of economic development	Per capita GDP	Yuan/person	+	[1]
	The proportion of secondary industry output	GDP of the secondary industry/Gross regional product	%	+	[38]
	The proportion of employment in the secondary industry	Number of employees in the secondary industry/total number of employees	%	+	[39]
	Secondary industry labor productivity	GDP of the secondary industry/number of employees in the secondary industry	Ten thousand yuan/person	+	[32,39]
Agricultural green development	The per capita disposable income of rural residents	The per capita disposable income of rural residents	Ten thousand yuan/person	+	[34,40]
	The level of agricultural mechanization	Total power of agricultural machinery/crop sown area	W/ha	+	[39,40,44]
	The rate of land output	Total agricultural output value/sown area of crops	Yuan/ha	+	[29,45]
	The level of farmland being irrigated	Effective irrigation area/arable land area	%	+	[39,44]
	The intensity of fertilizer used	Total fertilizer input/total sown area	Kg/ha	−	[29,44]
	The intensity of pesticides used	Total pesticide input/total sown area	Kg/ha	−	[29]
	The intensity of the used agricultural film	Total agricultural film input/total sown area	Kg/ha	−	[46]
	The proportion of disaster area	Infested area of disaster-stricken area	%	−	[34]

**Table 2:** Evaluation index weights of the coupling coordination degree of industrialization and agricultural green development.

Primary Indexes	Secondary Indexes	Index Weight
Industrialization	The level of economic development	0.2456
	The proportion of secondary industry output	0.2542
	The proportion of employment in the secondary industry	0.2522
	Secondary industry labor productivity	0.2481
Agricultural green development	The per capita disposable income of rural residents	0.1232
	The level of agricultural mechanization	0.1226
	The rate of land output	0.1236
	The level of farmland being irrigated	0.1247
	The intensity of fertilizer used	0.1260
	The intensity of pesticides used	0.1269
	the intensity of the used agricultural film	0.1272
	the proportion of disaster area	0.1258

Source: the authors calculated based on China Statistical Yearbook, China Industrial Statistical Yearbook, China Rural Statistical Yearbook, China Regional Economic Statistical Yearbook.

**Table 3:** China’s Industrialization Development Index.

Province	Years							Level	Sort
	1990	1995	2000	2005	2010	2015	2019		
Shanghai	0.8232	0.9440	0.9332	0.9200	0.6931	0.4710	0.4520	0.748	1
Tianjin	0.5903	0.6725	0.6926	0.8389	0.7618	0.7400	0.4009	0.6710	2
Jiangsu	0.3765	0.6579	0.5460	0.6748	0.6015	0.6925	0.7094	0.6084	3
Zhejiang	0.3490	0.7095	0.5572	0.6647	0.5993	0.6296	0.6061	0.5879	4
Guangdong	0.3314	0.6401	0.5500	0.6047	0.5669	0.5782	0.5178	0.5413	5
Fujian	0.2479	0.5189	0.4555	0.5076	0.5777	0.6499	0.6931	0.5215	6
Shandong	0.3320	0.5269	0.4583	0.5922	0.5959	0.6073	0.5017	0.5163	7
Liaoning	0.4668	0.5178	0.5415	0.4992	0.6075	0.5632	0.3775	0.5105	8
Beijing	0.6187	0.5994	0.5769	0.5455	0.3737	0.3026	0.2772	0.4705	9
Inner Mongolia	0.2606	0.2704	0.2902	0.4298	0.7215	0.7264	0.5121	0.4587	10
Xinjiang	0.3368	0.3215	0.4431	0.3903	0.5511	0.5541	0.5223	0.4456	11
Hebei	0.3389	0.4517	0.4182	0.4771	0.4534	0.5129	0.4150	0.4382	12
Shanxi	0.4133	0.4132	0.3689	0.4820	0.5394	0.3778	0.4011	0.4280	13
Hubei	0.2880	0.3663	0.4387	0.3317	0.4688	0.5169	0.4915	0.4146	14
Heilongjiang	0.4418	0.4848	0.5560	0.4992	0.4739	0.2797	0.1496	0.4121	15
Qinghai	0.4037	0.2770	0.2983	0.2956	0.4859	0.5737	0.4718	0.4009	16
Jilin	0.3114	0.3270	0.3693	0.3802	0.5341	0.5726	0.3076	0.4003	17
Henan	0.2150	0.3818	0.3040	0.3802	0.4847	0.5045	0.4768	0.3924	18
Chongqing	0.3122	0.4160	0.2557	0.2931	0.4909	0.4266	0.4978	0.3846	19
Shaanxi	0.2567	0.2707	0.2696	0.3016	0.4758	0.5249	0.5058	0.3721	20
Hunan	0.2981	0.2736	0.2553	0.3754	0.4050	0.4924	0.4448	0.3635	21
Anhui	0.2618	0.3914	0.2573	0.2692	0.4079	0.4774	0.4673	0.3617	22
Yunnan	0.3504	0.3520	0.2851	0.2780	0.3516	0.3815	0.5118	0.3586	23
Ningxia	0.3209	0.2730	0.2889	0.3122	0.4302	0.4239	0.4439	0.3562	24
Jiangxi	0.2084	0.2460	0.1992	0.3421	0.4355	0.5167	0.4680	0.3451	25
Sichuan	0.1381	0.2020	0.2557	0.2587	0.4353	0.4036	0.3345	0.2897	26
Guangxi	0.2103	0.3210	0.1947	0.2053	0.3410	0.4539	0.2951	0.2888	27
Gansu	0.2755	0.2704	0.2614	0.2501	0.3770	0.2932	0.2261	0.2791	28
Guizhou	0.2666	0.1886	0.1539	0.1461	0.2933	0.3702	0.3944	0.2590	29
Hainan	0.3902	0.2477	0.1109	0.1483	0.2177	0.1920	0.2704	0.2253	30
Tibet	0.0657	0.0371	0.1163	0.0611	0.1085	0.2725	0.4028	0.1520	31
the east	0.4423	0.5897	0.5309	0.5885	0.5499	0.5399	0.4746		
the middle	0.3047	0.3605	0.3436	0.3825	0.4687	0.4673	0.4008		
the west	0.2665	0.2666	0.2594	0.2685	0.4218	0.4504	0.4265		

Source: the authors calculated based on China Statistical Yearbook, China Industrial Statistical Yearbook, China Rural Statistical Yearbook, China Regional Economic Statistical Yearbook.

**Table 4:** China Agricultural Green Development Index.

Province	Years							Level	Sort
	1990	1995	2000	2005	2010	2015	2019		
Tianjin	0.7594	0.6894	0.6596	0.6249	0.6095	0.6375	0.6366	0.6596	1
Tibet	0.5309	0.6001	0.6323	0.6554	0.6192	0.6189	0.7097	0.6238	2
Zhejiang	0.5973	0.5611	0.5877	0.5480	0.6305	0.5624	0.5544	0.5773	3
Beijing	0.6847	0.5402	0.6998	0.5980	0.5829	0.4147	0.4870	0.5725	4
Shanghai	0.5969	0.4979	0.4131	0.4462	0.6377	0.5675	0.6539	0.5447	5
Jiangsu	0.5703	0.4752	0.4928	0.4864	0.5572	0.5673	0.5854	0.5335	6
Hebei	0.5682	0.5080	0.5180	0.5569	0.5424	0.5164	0.4841	0.5277	7
Hunan	0.5440	0.5178	0.5412	0.4500	0.5025	0.5152	0.4928	0.5091	8
Qinghai	0.5533	0.5271	0.4371	0.5261	0.5086	0.4381	0.4886	0.4970	9
Guangdong	0.5671	0.5049	0.5311	0.4184	0.5236	0.4472	0.4741	0.4952	10
Shandong	0.5479	0.4243	0.4432	0.4593	0.5032	0.4900	0.4612	0.4756	11
Xinjiang	0.5956	0.5061	0.4934	0.4465	0.4281	0.4067	0.4468	0.4747	12
Henan	0.5400	0.4087	0.4904	0.4616	0.4842	0.4801	0.4559	0.4744	13
Sichuan	0.5583	0.4571	0.4670	0.4256	0.4623	0.4811	0.4632	0.4735	14
Jiangxi	0.5608	0.5147	0.4285	0.4467	0.4764	0.4383	0.4325	0.4711	15
Anhui	0.5385	0.4050	0.4115	0.4109	0.4847	0.4676	0.4457	0.452	16
Chongqing	0.5131	0.5017	0.4508	0.3917	0.4448	0.4201	0.4413	0.4519	17
Ningxia	0.5388	0.4568	0.4252	0.4135	0.4536	0.4076	0.4616	0.4510	18
Inner Mongolia	0.5397	0.4667	0.4588	0.4447	0.4136	0.4063	0.4175	0.4496	19
Heilongjiang	0.5199	0.4442	0.4278	0.4423	0.4097	0.4407	0.4610	0.4494	20
Shanxi	0.5295	0.4704	0.4282	0.4086	0.4212	0.4456	0.3908	0.4420	21
Guizhou	0.4220	0.4623	0.4698	0.4144	0.3887	0.4516	0.4698	0.4398	22
Fujian	0.5066	0.4407	0.4163	0.4016	0.4410	0.4539	0.4053	0.4379	23
Guangxi	0.5323	0.4233	0.4500	0.3880	0.4006	0.4189	0.4093	0.4318	24
Hubei	0.5252	0.4347	0.3569	0.3421	0.4249	0.4532	0.4629	0.4286	25
Yunnan	0.5195	0.4544	0.4613	0.3728	0.3466	0.3725	0.3966	0.4177	26
Shaanxi	0.5121	0.4153	0.3992	0.3888	0.4029	0.3806	0.4240	0.4176	27
Jilin	0.5047	0.4292	0.3562	0.3873	0.3747	0.3898	0.3829	0.4035	28
Hainan	0.5588	0.5146	0.4840	0.2773	0.3246	0.2643	0.3441	0.3954	29
Liaoning	0.4444	0.3857	0.3898	0.3903	0.3489	0.3771	0.4205	0.3938	30
Gansu	0.4881	0.4330	0.4218	0.3523	0.3585	0.3062	0.3112	0.3816	31
the east	0.5820	0.5038	0.5123	0.4734	0.5183	0.4817	0.5006		
the middle	0.5322	0.4543	0.4390	0.4312	0.4454	0.4523	0.4403		
the west	0.5194	0.4694	0.4525	0.4263	0.4354	0.4296	0.4546		

Source: the authors calculated based on China Statistical Yearbook, China Industrial Statistical Yearbook, China Rural Statistical Yearbook, China Regional Economic Statistical Yearbook.

**Table 5:** Global Moran index of coupling coordination degree of China’s industrialization and agricultural green development in some years.

Year	I	E (I)	Sd (I)	Z	*p*-Value
1990	0.169	−0.033	0.072	2.815	0.002
1995	0.224	−0.033	0.072	3.588	0.000
2000	0.296	−0.033	0.074	4.452	0.000
2005	0.378	−0.033	0.074	5.522	0.000
2010	0.245	−0.033	0.074	3.765	0.000
2015	0.079	−0.033	0.072	1.562	0.059
2019	0.143	−0.033	0.074	2.390	0.008

## Data Availability

Publicly available datasets were analyzed in this study. This data can be found here: China Statistical Yearbook (http://www.stats.gov.cn/tjsj/ndsj/ (accessed on 25 June 2021)), China Industrial Statistical Yearbook (https://data.cnki.net/trade/Yearbook/Single/N2021020054?z=Z012 (accessed on 25 June 2021)), China Rural Statistical Yearbook (https://data.cnki.net/area/Yearbook/Single/N2010080069?z=D22 (accessed on 25 June 2021)), China Regional Economic Statistical Yearbook (https://data.cnki.net/trade/Yearbook/Single/N2012030056?z=Z030 (accessed on 25 June 2021).
